# Memoirs of the Royal Academy of Medicine. Vol. X

**Published:** 1843-10-01

**Authors:** 


### Medico-Chirurgical Review,

### No. LXXVIII.

[No. 38 of a Decennial Series.]

JULY 1, TO OCTOBER 1, 1843.

### Mémoires de l'Académie Royale de la Médicine. Tom. X. Paris, 1843.

### Memoirs of the Royal Academy of Medicine. Vol. X.

Although the present volume, of what may be termed the Medico-Chirurgical Transactions of France, does not contain any of the elaborate and lengthy dissertations which have characterized most of its predecessors, it abounds in a far larger proportion of useful practical papers. Abstracts of most of these we will present to the reader.

### I. On Super-Malleolar Amputation. By MM. *Arnal* and *Martin*.

The authors apply this term to amputation of the leg when performed at about three fingers' breadth above the malleoli. The object of the paper is to prove that this site is every way preferable to that usually chosen, viz. five fingers' breadth below the knee. It is so from the greater facility with which the operation is there executed—the less degree of pain that is caused, and consequently less subsequent fever, re-action, and their results—the avoidance of the great volume and projecting angle of the tibia, the difficulty of covering the broad stump this causes, besides the danger of inflammation and caries of the bone, and the risk of producing a conical stump—the less hæmorrhage to control, fewer ligatures to apply, and less fear of secondary bleeding—the less disposition to spasm in the stump—and the greater facility of effecting union by the first intention. The objections raised to this operation are without foundation. Thus, the greater number of tendons implicated, accompanied as they are very low down with muscular fibres, does not give rise to the greater probability of gangrene or delay in healing the stump—it is not a fact that large collections of matter are subsequently formed, amid the connecting cellular tissue, which is here actually in less quantity than higher up—it is erroneous to say that a small stump, covered only with skin, is less solid and more liable to secondary inflammation than a larger one, covered with muscle; for, actually, in this latter, all the soft parts, excepting the skin, become absorbed, or converted into a kind of ligamentous substance. Larrey has much exaggerated the predominance of the flexor muscles after this operation, for the flexion which does exist, arises, not from this, but from the use of faulty apparatus, requiring constant flexure of the knee-joint. After the operation, the limb should be kept extended, and thus its proper position will be secured, which can only otherwise be produced by time and exercise. When a proper apparatus was used, this forcible flexion, so much spoken of, was not observed in any one of 97 cases alluded to by the authors; nor have any of these suffered from compression of the nerves of the stump—an accident said by some to follow this description of amputation. The results of these 97 operations are presented in a tabular view. From this, the mortality is seen to be but 1 in 10, while the experience of the ordinary operation at the Parisian Hospitals, as stated by Dupuytren, places the mortality at 1 in 4, or, according to our authors, at 1 in 3. The period occupied in the cure is also about half that required after the ordinary operation.

The fact of this operation having been adopted and abandoned by several surgeons, is explained by the authors to have arisen from the non-existence of any properly-contrived artificial leg. It is an extraordinary fact that, although some monuments represent persons wearing wooden legs, no ancient medical author enters into any description of these or other apparatus calculated to supply the loss of the extremity. It is from Ambrose Paré the first ideas respecting these are derived; but those described by him are too complicated and ingenious to have been other than the results of repeated experiments and modifications; and indeed, few, if any essential, improvements have been made from that period to the present. The authors describe the false leg they would wish to substitute for those in use, but we must refer our readers to their paper for this, as any abridged description would be unintelligible.

### II. A New Mode of relieving Prolapsus Ani. By *A. Robert.*

The author having stated the most powerful cause of procidentia ani to be the paralysis or relaxation of the sphincter, and having enumerated the modes hitherto adopted of relieving the affection, thus proceeds.

> "It is easy to observe that if these modes of treatment differ among themselves, they have one common result, that of forming, either above or below the sphincter, a wound, either with or without loss of substance, which in suppurating and cicatrizing contracts the anus, and determines a more intimate union of the intestine or the skin with the subjacent parts. In ordinary cases this cicatrix resists sufficiently well, because it receives a solid support in the sphincter ani, which has lost little of its contractile power. But if this muscle is itself affected with extreme relaxation, whether primary, or the result of the long and excessive distention which it has been submitted to by the tumor, it is easy to foresee, that the cicatrix, being no longer supported, will become extended, and the prolapsus reproduced."

M. Robert next relates a very bad case of prolapsus ani of considerable duration occurring in a washerwoman, as an example of his mode of relieving the affection. He made two incisions at the posterior border of the anus, in the form of a V, the apex meeting at the coccyx, and, having removed a corresponding portion of the sphincter, he united the parts by the suture, the threads of which were removed on the 6th day. On the 15th day (constipation having been induced by abstinence and opiates) fæcal matters were removed by a scoop, and on the 18th day the woman had a natural stool without the reproduction of her infirmity. She became eventually completely cured.

### III. An Efficacious Means of relieving Hæmorrhage, occurring after the Lateral Operation for the Stone. By M. *Begin.*

The only means of any efficacy employed hitherto against this formidable and not unfrequently fatal occurrence are the ligature and torsion, for compression can seldom be borne long enough, or properly adapted to the site of the hæmorrhage. The inability of detecting the bleeding vessel has also repeatedly rendered both the ligature and torsion unavailable. The author recommends, having practised it with success, the constant and copious *irrigation* of the parts with cold water. Of the two cases he narrates, in the first this was accomplished by means of the ordinary lavement syringes, employed vigorously by the pupils of the hospital—one constantly filling, while the other was working. The surface of the wound and all the neighbouring parts were in this way freely played upon for some hours without intermission. In the other case, an apparatus was contrived to admit of a constant current of water being directed upon the parts. In both, most complete success attended the practice, although the patients were reduced to an almost hopeless state prior to its institution. The irrigation seemed also to produce a very sedative effect, and was not followed by any re-action of an inflammatory character. The apparatus required is simply a caoutchouc tube and elastic canula, the patient being brought to the edge of the bed during its use.

M. Begin suggests the extension of this mode of treatment to some of the varieties of uterine hæmorrhage.

### IV. The Temperaments considered in their Relation to Health. By M. *Royer-Collard.*

The author, who is Professor of Hygiene in the Faculty of Medicine, expresses his regret that, amid the great progress of medical science, the department of hygiene should continue stationary, and unfounded upon any true physiological basis. He commences his paper by stating, the ordinary descriptions of the temperaments are founded upon mere conjecture. Thus, (*e. g.*) the statement of the *sanguine* temperament being characterized by the possession of a greater quantity of blood, greater rapidity of circulation, a more developed condition of the circulatory organs, and a greater activity of respiration than other temperaments, is quite destitute of proof; for not only are experiments confirmatory of it wanting, but it is not corroborated by the result of post-mortem examination. So, too, the preponderance of the nervous system, assumed as characteristic of the *nervous* temperament, may be associated with any of the others. That state of the system, also, termed the *bilious* temperament, is in nowise proved to be dependent upon the presence of an excess or faulty distribution of the bile. That this is the case chemical analysis fails to show, while the morale of such subjects is dependent upon peculiarities of cerebral rather than of abdominal organization. The symptoms of disease, have often, by a forced analogy, been transferred to the explanation of a temperament.

"I believe I have shewn how far the generally received doctrine of temperaments is from presenting that certainty which should characterize a physiological doctrine. All in it is vague; mere suppositions in the place of positive observations; analogies sometimes ingenious, but almost always destitute of proof: and, lastly, an almost utter neglect of all the discoveries which chemical analysis has furnished us with in recent times—such is the condition in which the doctrine remains. What do we see in the usual expositions of the various temperaments. Two things are indicated. First the external signs, which serve to distinguish them from each other; and 2nd, the organic cause, to which they are referred, and by which they are explained. But what an explanation! Does it furnish demonstrated facts which may explain others? Nothing of the kind! Mere suppositions whose uncertainty I have shewn. Evidently this is no logical procedure; this is not that severe inductive process, the only true instrument of discovery, introduced by Bacon and Newton into the study of physical phenomena, and without which all theory is inconsequent and baseless." 151.

A temperament is an universal condition of the economy, one of the forms or varieties in which the condition of health may exist in; and its source and conditions are not to be sought for in this special fluid or that solid, but in circumstances influencing the entire economy. The *blood*, or the nutritive fluid of the body, and the *nervous fluid* (so to speak) are the two universal principles of the body, modifying its condition, whether in health or disease, by their double influence; and it is to the various manifestations offered by these we are to look, not for the *cause*, but for the *characters* of temperaments.

It is, especially, from the results of the chemical examinations of the blood, which have been made of late years, that the author considers himself in a condition to propose a better explanation of the temperaments; and it is from the pursuit of similar examinations he hopes for further improvements. The average composition of the blood as determined by Lecanu, Andral, and others is

| | |
|---|---|
| Fibrine | 0·003 |
| Globules | 0·127 |
| Solid organic matters | 0·072 |
| Inorganic matters | 0·008 |
| Water | 0·790 |

But while these are admitted to be the average relative proportions of the elements of the blood, it is also known that these proportions may, and do, vary, consistently with the state of health, within certain limits. Although the researches of science are not yet sufficiently exact to point out these limits with precision, and to demonstrate where the condition of health terminates, and that of disease commences, some general conclusions of importance may be arrived at. Thus it is found that the proportion of colouring matter and globules designates the degree of vigour of the individual. Andral has in this way observed such difference as regards strength of constitution, but not in reference to disease. In plethora, or the fullest state of activity of the nutritive functions, he found the proportion raised from 0·127 to 0·140. In the lymphatic temperament, where there is debility, the proportion is lower than the average. The proportion of fibrine seems to undergo neither increase or diminution, unless actual disease is present. The aqueous portion of the blood is found in larger proportion in debilitated constitutions, and in less in those which manifest power.

The blood cannot be identical in its composition in all parts of the economy of even the same individual, for its condition on entering and leaving a secretory organ must be very different. Chemistry enables us to fully appreciate the change it undergoes in traversing the pulmonary vessels; and the same thing occurs, in different degrees, in its passage through the structure of other organs. The changes which are effected by means of the various secretory organs, not always occurring alike in different individuals, may give rise to modifications in their temperaments. In reference to the bilious temperament (*e. g.*) the author observes—

"There are cases in which a primitive modification of the secretory powers of the liver may give rise to the biliary temperament of authors. This happens, when from any cause this organ does not extract from the blood the materials of the bile, in sufficient quantity for the necessities of the system. It will be seen that there must then predominate in this fluid too large a proportion of carbonized, and hydrogenous elements, and of fatty, coloring, &c. matters, which should have been separated by the biliary secretion. But other causes may produce the same effects, for these matters are not only eliminated by the liver in the form of bile, but are so, also, by the lungs in the form of water and carbonic acid, by the kidneys, the skin, and various other modes of elimination. Now, if any accident occurs to impede this elimination, hydrogen and carbon will be found to superabound in the blood. This superabundance, and the consequent predominance of venous over the arterial blood, the slower venous abdominal circulation, and the congested condition of all parts of the system where it prevails, form the essential characters of what has been called the bilious temperament." 160.

If the condition of the *blood* offers the truest material expression of the temperaments, that of the *nervous system* must not be neglected; for, if the one is the centre of the vegetative life, so is the other the centre of the animal life; and the two have a reciprocal influence on each other. But, as before observed, what has been called the nervous temperament may unite itself with very different conditions of the economy. Of the influence of the nervous action he offers the following illustration.

"Take two men, possessing in the highest degree the sanguine temperament, as described by authors, and predisposed to cerebral congestion. One of these will not be able to bear an elevated temperature. During the heat of Summer, or in a heated apartment, he will suffer much from the afflux of blood to the head; and will become relieved upon a diminution of temperature. The other will prove effects directly the reverse under the same circumstances. Why this difference, each having the same temperament? Because, in the first case, the circulation is slow, and the peripheral exhalation scanty; while in the other, the circulation is active, and when the temperature is high, the cutaneous and pulmonary transpiration is abundant, and the central organs are quickly relieved. But in a cold atmosphere the blood is repelled to the interior, and visceral congestions soon follow. Do you prescribe for these two men, the same regimen, merely because they are said to possess the same temperament? If so, one of the two must suffer. Is it not evident we must here take into account something besides the external characters of the temperament, or the constitution of the blood, and that the so opposite circumstances observed in the two depend obviously upon the nervous action, which is entirely different in the two cases." 164.

The author declines offering any arrangement or nomenclature of temperaments, satisfied with having pointed out the erroneous ideas prevailing upon the subject, and the mode in which future investigations should be conducted.

Of the distinction to be drawn between *temperament* and *constitution*, he thus speaks.

"*Temperament* is essentially variable: age alone may suffice to substitute one temperament for another; so, also, may hygienic agents, climate, professions, manners, habits, &c. &c. It is not so with the *constitution*. Every man is primarily endued with a particular constitution, distinct from temperament, and in the study of which the doctrine of hereditariness essentially enters. The constitution may be modified by regimen, but not destroyed. In a word, the constitution is the foundation of the individual being, the temperament is its more or less durable form of existence." 168.

### V. THE SUPPLY OF FOOD IN RELATION TO DISEASE AND MORTALITY. By *F. Mélier*.

The Anti-Corn-Law-League might print this paper among their Useful Knowledge Tracts. The author enters into a long examination of the prices of corn, the prevalence of disease, and the amount of mortality in the eighteenth and nineteenth centuries; and finds that high prices and a high ratio of disease and mortality have ever gone hand in hand. From 1815 to 1835, the population and produce of the harvest each increased just 12 per cent. The influence of the price of corn, however, has become less visible of late years, owing to the increased cultivation of the potato and garden produce contributing to supply the deficiences of bad years.

### VI. ON THE VALUE OF THE MICROSCOPIC EXAMINATION OF THE MILK IN THE CHOICE OF A NURSE. By *Alph. Devergie*.

M. Devergie, having the superintendence of the public establishment for nurses, took the opportunity of testing M. Donne's researches respecting the microscopic examination of the milk. The following are his conclusions:—

Under the microscope, a nurse's milk presents globules of three different sizes, that which contains the largest being the most nutritious. Middle-sized globules are most generally observed. The milk with large globules (which, however, the stomachs of some infants will not bear) is usually, but not exclusively, found in the sanguine-lymphatic temperament. The number of globules (or richness of the milk) is not always proportioned to their size. Large globules are sometimes found in nurses whose external appearance is unfavourable. There may be considerable difference in the milk in the two breasts, often arising from the habit of suckling especially from one breast. When the difference is very great the nurse suckles exclusively from one breast.

The recommencement of suckling after a temporary suspension is attended with an increased richness of the milk. The age of the milk, the age of the nurse, or the colour of her hair, are not attended with corresponding microscopic appearances. Breasts of a middling size furnish the best milk.

He thus concludes his paper.

"A good nurse should be a woman from 25 to 30 years of age, strong in constitution, having a broad chest, sanguine-lymphatic temperament, brown hair, white and undecayed teeth, complexion, and lips coloured. Her breasts should be pyriform, with clearly-defined nipples, and without the veins being much dilated, or the areolæ too large. The milk, when placed in a spoon, should be white with a bluish tinge, not too thick, and of a sweet taste. Examined by the microscope, the size and number of the globules will be seen. But when satisfactory in this respect, it may not agree with the child if its assimilative powers are not equal to milk of the character observed. If its health suffer in the experiment, the microscope may be very useful in ascertaining the differences presented by the milk of a new nurse. But there are alterations in the condition of the milk of which we know nothing, and whose nature the microscope does not elucidate." 222.

### VII. On Epidemic Cerebro-Meningitis and Cerebro-Spinal Meningitis. By M. *Rollet.*

These affections have raged, at different intervals, for several years, among the chief garrisons of France—civilians in the same towns, though not altogether exempt, being rarely attacked. Most authors have considered the two affections as being but different degrees of the same disease, but M. Rollet thinks differently, and arranges the cases he has seen in two categories. 1. Those (cerebro-spinal meningitis,) with or without affection of the intellectual faculties, but without any lesion of sensibility or motion; and, 2, (cerebro-meningitis,) those manifesting affections of the intellect, the sensibility and motion, with more or less complete abolition of the functions of the external senses.

He relates 28 cases. All his patients were of the sanguine temperament, and consisted, for the most part, of new soldiers, hitherto unaccustomed to the labours of the camp. The affection occurs especially in Spring and Summer.

We need not enter into a description of the symptoms; and the post-mortem appearances chiefly consisted in the altered condition of the pia mater—effusion into the arachnoid, or cerebral ramollissement also sometimes occurring.

Antiphlogistic treatment, of a vigorous character, usually proved useful when promptly applied. When, however, the state of irritation had persisted some time, a condition resembling compression of the brain, attended with complete depression of powers and a filiform pulse, frequently succeeded. The object now is to relieve the oppressed brain by exciting re-action upon the surface of the body, and thus direct the blood from the centre to the periphery of the system. But ordinary means of counter-irritation produce no effects upon the skin. The author institutes the following sufficiently vigorous procedures *simultaneously*. The patient having been laid on the belly, from six to eight slight applications of the actual cautery are made along each side of the spinal column; large sina-pisms are applied to the feet, and kept constantly hot with boiling water, while an ammoniacal pommade is rubbed into the inner parts of the thighs and legs, and cupping-glasses are applied to the back of the neck. The re-action usually commences in about an hour, and when it is sufficiently established, bleeding must be practised in proportion to the strength of the patient, applying at the same time cold to the head, and heat to the feet.

Certainly nothing but the desperate condition into which the author re-presents these patients to have been reduced could justify this treatment, which, he says, he has found very successful.

### VIII. Account of the Epidemic Miliary Sweat, which prevailed in the Department of Dordogne in 1841. By *H. Parrot.*

Various provinces of France, especially Picardy, have at different periods been affected, on a small scale, with these sweating sicknesses, which some centuries back committed such ravages in our own country. They have usually been attributed to the miserable physical condition of the people in the districts in which they prevail, as shewn in the bad habitations, want of means of removal of filth, damp, &c. and the use of bad black bread, chesnuts, &c. &c. with an almost utter deprivation of animal food and wine—the French Peasant's beer. Dr. Parrot states that all these circumstances prevailed in the district whose devastation he reports upon; but he seems to doubt how far these causes may have been operative in the production of the disease, inasmuch as its progress frequently took place in a contrary direction to that in which filth and want chiefly pre-vailed, while the lowest dregs of the population were among those who chiefly escaped. He attributes considerable influence to meteorological causes, and geological peculiarities.

The epidemic continued for five months, manifesting itself in different districts in five separate invasions. It frequently caused death in a very rapid manner, and that so insidiously, that many cases which seemed to be so slight as hardly to call for notice, were among the most fatal, so that, at last, it was laid down as an axiom, that a cure could never be promised. In many patients profuse sweating, followed sooner or later by vesicular eruption, seemed the sole effects of the disease. The health and strength, and especially the appetite, continuing good. In other cases the sweating was profuse beyond conception, accompanied by a peculiar odour, and followed by the development of severe cerebral symptoms: tongue was usually moist but yellow, and the appetite, even in bad cases, urgent beyond constraint. Pulse in bad cases hard, vibratory, and frequent. Epigastric pulsation a frequent symptom. Blood drawn presented softened coagulum and no buffiness. Post-mortem examination revealed no important lesions sufficiently explanatory of the mortality. Out of 597 cases observed by the author, 321 were women, and a remarkable feature of the disease was its inducing menstruation in women, even prior to their proper periods, and not suppressing it when present. The extraordinary number of menstruating women affected, and the great number of abortions which occurred, justly entitled the affection to be considered a powerful *emmenagogue*. Persons of robust constitution seemed most liable to attack, and when seized with the disease fared worse than their feebler neighbours. The *Sulphate of Quinine* (the fever accompanying the disease generally assumed a remitting form) was found to be a powerful means of cure. Accessory means, such as nitrate of potass, venesection, forbidding the excess of covering assumed by the patients, and a due regard to frequent changes of linen, were had recourse to.

The population of the affected district amounted to 83,342: the number of persons affected to 10,805; and the deaths to 797.

### IX. On Formative Hygiene (Organoplastie Hygienique). By M. *Royer-Collard*.

The object of Hygiene should not be merely the preservation of health by the prevention of disease; but, also, by the amelioration and perfecting the instruments of life, enable the organism without danger to develop the entire amount of power of which it is possessed. This second object has been much overlooked, and we are at a loss to point to the rules or principles for its accomplishment. That this is the case has always surprised the author; and he asks the following question—

"Since hygiene can always, by the aid of regimen, moderate or excite the vital action, increase or diminish the strength, and direct to a certain degree all organic operations, to what point, also, might not a well considered and systematically combined system of regimen effect a modification in our organs by means of nutrition, and form them as it were as we wish, to develop such a part, to diminish or obliterate another, to change, in fact, artificially, if not the essential constitution of the body, at least its most variable forms which we have been accustomed to term its temperaments?

"Nor is there anything so unreasonable in this *à priori*. Regimen, in fact, comprehends five principal things,—diet—conditions of the atmosphere—exercise—the generative functions and moral influences. That each of these exerts a powerful influence is known to every one; so that we can very well see the possibility of obtaining results which have been foreseen or calculated beforehand, by means of a regimen in which we can rigorously specify the choice of the material of nutrition, and the direction of the vital powers.

"Anatomy and physiology have made no remarkable progress for these twenty years, but by the comparative study of man and other organised species. Pathology has scarcely entered upon this path. Hygiene is yet more backward in this respect than any other branch of medicine, and, nevertheless, more than any other, she might find in such a study precious and multiplied facts. The practice of agriculture, the rearing of cattle, and the training of domestic animals, have amassed during ages a treasure of positive observations and experiments ready made. The smallest farmer of our fields possesses ideas in which we are deficient

—a curious assemblage of truth and error, the rough result frequently of a gross empiricism, but sometimes also the result of the inspiration of genius." 479-81.

It is principally in the vegetable world that man exhibits the power he has acquired over nature. The modifications which various plants undergo by varieties of culture, and the surety with which these may be produced, are too well known, and have been too well illustrated by Liebig, to call for farther notice here.

Among insects, as in the case of bees and ants, we find nourishment of peculiar kinds or quantities conferring organic powers which did not heretofore exist. Edwards has been able, by exclusion of light and air, to prevent the tadpole, not increasing in size, for that it does, but still more surprising, undergoing its natural transformation into the frog. The wonderful effects which have been produced in England since the time of Bakewell, by the feeding and crossing the race of the various domestic animals, are almost incredible.

" For fifty years," says our author, " have the ideas of Bakewell now prevailed throughout Europe. The art of regimen has been carried to an astonishing perfection. It is well known now, by certain signs, which animals are proper, which improper for fattening, what conditions are necessary to bring them to a certain determinate size, what organs must be directly acted upon to increase nutrition, which description of food produces muscles, which fat, milk in cows, wool in sheep. For each animal is measured its exact proportion of food, air, and light, and the degree of exercise that is necessary to bring it into such or such a condition, for rendering it fitted for such or such a state. It is known when the fat accumulates especially under the skin, in the great cavities, or in the tissue of the organs. The number of pounds to be acquired daily during the treatment may be accurately specified. Every description of animal has been submitted to this fattening system; and thus even fish, which have been castrated, are placed in wet moss, and remaining there motionless, living only to eat and digest, acquire a most extraordinary size." 489.

Seeing the manner in which we may trace the influence of regimen through the entire zoological series, we should be quite justified in declaring its applicability to man. But positive facts proving this exist in abundance; for, not only do we, on the one hand, see the effects of a faulty regimen in the production of disease and degeneration, as in rickets, scrofula, and the stunted condition and susceptibility to disease in the lowest classes; but we also find that, where regimen is carefully attended to, as in the case of training, the athletic powers of endurance and longevity of the boxer, runner, &c. are produced. The author enters at some length into the consideration of the principles and results of training, which are too well known to the English reader to require being reproduced here, and then proceeds.

" These short explanations will suffice to shew physicians what is accomplished in general by *training*. Nothing can be more simple than such a regimen; and, I may add nothing can be more physiological. It is an exact application of the famous rule of the Methodists, as delivered by Cælius Aurelianus. '*Recorporativis utendum viribus, ita ut, resectis vitiosis carnibus, ac renascentibus novis, reformata organa redeant ad sanitatem.*' The Methodists acted as the trainers. They first bled and purged, and then prescribed wholesome food and exercise. Can we be astonished then at the results of training? We should rather feel astonished at our own surprize, and that a practice so reasonable should appear to us as something extraordinary and incredible. We should feel astonished that physicians, by the aid of science and often of scientific subtilties, should have so far wandered from the straight and natural path, as to require to be brought back to it by ignorant empirics who have contented themselves with the roughest reasonings, supported by numerous and positive experiments.

"After having accumulated this evidence, which I could easily increase, I may be allowed to establish as an incontestable fact the great power of this art, which, seizing hold, in some measure, of the system of nutrition, directs it methodically towards a determined end, and changes the intimate structure of organs, sometimes in one manner, sometimes in another, without employing any other means than regimen. This principle once laid down and properly understood, who does not see at a glance the great use that may be made of it. How many forms or different degrees of health might be advantageously modified by a systematic regimen, which should exact, on the one hand, but an active and intelligent superintendence, and, on the other, patience and submission to its rules? How many morbid conditions are there also, against which therapeutics exhaust, oftentimes inopportunely, so many powerless or dangerous prescriptions!

"My object, to day, has been to present but the first part of my researches, and that in a very abridged form. The second, which I regard as the more important, will be purely scientific. In it I shall submit each fact to a thorough examination, and deduce thence its true value and the extent of its application." 498-500.

### X. On Syphilitic Eruptions. By Dr. *Gibert.*

From among the author's preliminary remarks we extract some observations on *Gonorrhœa.* Of 60 cases of syphilitic eruptions, there were 14 cases in which gonorrhœa was the only symptom which had preceded them, and 28 others in which it was one of the primary symptoms. Dr. Gibert considers, with B. Bell, that the principal site of gonorrhœa is the fossa navicularis, and, with Morgagni and Hunter, that it exists independent of ulceration. Without absolutely denying the correctness of Ricord's assertion, that secondary symptoms in gonorrhœa always proceed from a concealed chancre, he thinks at present it is destitute of the necessary proofs.

In *women* the chief seat of the disease is the *meatus urinarius;* but, whenever he has used the speculum, the author has observed a copious discharge also issuing from the lips of the os uteri. This uterine discharge continues long after all other has been relieved, and is capable of communicating contagion, even although it resembles mere common leucorrhœa. In reference to examination by the *speculum,* the author thinks it necessary to caution his readers, that a *granular erosion* of the cervix uteri, which is sometimes met with, associated with secondary symptoms, is not to be mistaken for a primary chancre of this part, which is a much more rare affection. A granular erosion of a simple inflammatory character is not always easily distinguished from one of syphilitic origin. Moreover, the mucous membrane of the cervix is sometimes excoriated, reddened, or aphthous, and this part may, in women who have borne children, offer great varieties as to length, volume, situation, &c.

which have too often been regarded as morbid; and the author justly criticises the practice of those, who, in slight and insignificant affections of the part, (whose very existence is ascertained not by symptoms but inspection, and which let alone would disappear) order leeches to the cervix, bleed, cauterize, insist upon the recumbent posture, &c. He alludes to cases where this had been done even by practitioners of great repute.

*Urethritis* then is the characteristic symptom of gonorrhœa in woman, and to term the disease vaginitis, as has been done by some who have examined the cases superficially, is erroneous. Of 216 cases of discharge, supposed to be of a venereal origin, in the Hôpital de Lourcine, in 88 discharge was found to be proceeding from the urethra, and in 40 only was there any indication of vaginal affection. But this last figure should be yet farther reduced, for, in many cases, a slight redness only was found, which repose soon dissipated, while, in others, a slight milky discharge only combined with the proper discharge. Those cases in which urethritis was not observed had not been admitted until a month or so after the gonorrhœa had first appeared, at which period the urethral discharge usually ceases, that from the uterus continuing for a longer one. The difficulty of diagnosis is great, for the discharge may continue contagious, when only a small quantity exists at the os uteri, and infection has been imparted when all appearances of discharge have ceased. The same thing it is well known occurs in men, who have infected their wives, after having been pronounced cured by experienced surgeons.

The author arranges the syphilitic eruptions into eight species, corresponding with the eight orders of cutaneous affections he has established in his work upon that subject.

| | | |
|---|---|---|
| " 1. | Exanthemata | (Roseola syphilitica.) |
| 2. | Vesicular | (Eczema and Varicella S.) |
| 3. | Bullous | (Rupia S.) |
| 4. | Pustular | (Ecthyma and Acne S.) |
| 5. | Tubercular | (Multiforme and Lupus S.) |
| 6. | Squamous | (Lepra and Psoriasis S.) |
| 7. | Papular | (Lichen S.) |
| 8. | Macular | (Venereal Spots and Stains.)" 527. |

These, however differing among themselves, offer the following characters in common, distinguishing them from non-venereal eruptions:—1. Their copper-coloured appearance, of every shade from a violet-red to an earthy yellow. 2. Their great diffusion, they, with some exceptions, tending to spread over the entire surface of the body. 3. Their obstinate persistence when no remedies are applied, and their ready yielding to these when appropriate. 4. The peculiar appearance of the spots, and cicatrix they leave on subsiding. The author has found the proto-iodide of mercury and the deuto-chloride of mercury the most powerful internal remedies; and the cases which did not quickly yield to these were advantageously treated by mercurial inunctions, applied to the arm-pits at bed-time, and retained there during the night. Local applications, although subsidiary, were not neglected, and were chiefly formed of ointments of the abovementioned preparations. Of 100 cures which were accomplished, there were—

| | |
|---|---|
| In one month .. .. .. .. .. | 44 |
| In six weeks .. .. .. .. .. | 28 |
| In two months .. .. .. .. .. | 18 |
| In three months and more .. .. .. | 10 |

### XI. On Intermittent Affections, with Short Periods. By Dr. *Mélier*.

Perhaps it will be better to commence our notice of this very useful paper by transcribing the conclusions the author has arrived at.

After referring to the division of diseases into continued and intermittent, and having dwelt upon the practical utility of recognising intermittence wherever it is to be found, he says—

"1. I have endeavoured to establish—That the types generally admitted, as the quotidian, tertian, quartan, &c. are not the only ones. 2. That to these we must add others, much shorter, abridgements of the longer, as it were; and without attaching that importance to the title which alone belongs to the thing itself, I propose to call these *intermittent diseases with short periods*. 3. That the diseases which observe these rapid alterations present the same indications as the ordinary intermittents, and require, like them, the employment of the sulphate of quinine.

"It is remarkable that, while authors have sought with much care to establish the existence of intermittents with longer periods than those generally admitted, such as quintans, sextans, &c. which are all more or less problematical, or at all events very rare, they have not inquired whether shorter intermittents might not exist: scarcely, indeed, does any author even mention a bi-quotidian type. And, yet, when a disease manifests itself several times daily by well-marked characters, and after having continued for a certain period (whether always the same or not) ceases during a given time, one or two hours, for example; then returns, again to cease, and that in three, four, or five different attacks, always distinct, and separated from each other by marked, though perhaps unequal, intervals, how must we characterise it. Is it not merely a kind of intermittent, but a real intermittent.'"

Dr. Mélier first applied these ideas, a few years since, to the treatment of a case of *infantile convulsion*. It occurred in a child six weeks old, and had resisted every means of relief for 48 hours. Death seemed inevitable, when the author, observing there were marked intervals in the progress of the affection, recommended one-grain doses of quinine every hour. The success was complete. Since that period he has accumulated a great number of facts confirmatory of this practice, and many of the best practitioners of Paris have furnished him with corroborative evidence.

"For these six years past, when the first case occurred, a great number of facts observed in reference to this point have confirmed me in my ideas, and in the propriety of the practice founded upon them. At present, whenever I am called to an infant affected with convulsions, (I do not speak of those which are purely symptomatic, and depending upon some obvious local suffering, as indigestion, teething, &c. offer a special means of relief—but I mean convulsions usually called, for want of a better term, essential, *i. e.* arising from a disordered state of the nervous system, independent of obvious lesion)—if these occur, as is usually the case, by crises or attacks, separated by longer or shorter intervals of repose, I then look upon the affection as an intermittent, and whether the interval consist of but one, or of several hours, I give the quinine, and I can declare truly, that I have often been enabled to arrest the disease in the most ready and unhoped-for manner. I am convinced that I have, in this way, saved the lives of many infants, who, treated in another manner, seemed to me destined to certain death.

"It has been remarked, by many authors, that bark and its various preparations never succeeded better than in those cases, in which the access of the paroxysm was marked by distinct shivering, or at least coldness. Now, if an infant, who is about to suffer from the convulsive attack, be observed, I will not say that a true shivering takes place, but there is certainly, in many cases and in different degrees, that precursory spasm so well described by Cullen, that concentration upon the internal organs which precedes a severe re-action. The infant becomes pale, its lips are discoloured, and its cheeks frequently chilled; and then comes on the muscular action characterising convulsion. Does not this progress of the symptoms, which I have more than once observed, authorize a far greater analogy between certain convulsions of infancy and intermittents, properly so called, than I have sought to establish; and does it not furnish yet another motive for employing the quinine?" 560-2.

Dr. Mélier alludes to the observations of MM. Piorry and de Lens, which were made even prior to his own, although not founded upon the same reasoning. M. Piorry recommends the quinine in cases of cephalic irritation in infants, believing that mere alternation of redness and paleness of the cheeks furnishes a sufficient indication for its employment. But Dr. M. states, that the perusal of the cases adduced proves to him that good only resulted from its use in those of them in which intermittence, however short, was present. M. de Lens, who has long employed the quinine, in a greater variety of affections than most persons, furnished the author with a statement that the majorty of febrile affections offer intermittence at their commencement, and that, if at this period quinine were administered, a cure might forthwith be effected. The obscurity of the interval usually prevents its recognition by the practitioner, and the affection then becomes established and continuous, and incapable of relief by this medicine. He says, similar observations may be made regarding many of the cerebral and convulsive affections of children, diseases of women, originating in distress of mind, &c., in many affections of an ambulatory character, as some cases of gout, rheumatism, neuralgic pains, and visceral phlegmasia. In many of these cases, he adds, in which the intermittence is very doubtful, the quinine is usually harmless at best, and often very serviceable.

Dr. Mélier has found this medicine also applicable to cases of eclampsia, obstinate hiccough, neuralgia of the uterus, rectum, and other viscera. Intermittent neuralgiæ, of the ordinary character, have been long successfully treated by its means, but this has not been the case when the affection has returned at the short intervals here described. He thus concludes:—

"I will not relate any more examples, although I could easily furnish them. Nervous affections are not the only diseases which present them, for they may be found in fevers, hæmorrhages, &c. Hooping-cough, if I mistake not, has also furnished them. I believe, too, I have said enough to establish the fact, that, besides the intermittent affections generally admitted to exist, there are others, with short or abridged types, of the same nature, and presenting the same indications.

"I see intermittence whenever I see an obvious alternation of symptoms and cessation, however short the intervals may be. But in proportion as the attacks approach each other more nearly, it is obvious that the short intervals which separate them must be more actively employed, and carefully seized. The maxim of the Father of Medicine, *Occasio præceps,* was never more applicable than here.

"Moreover, intermittent affections with short periods, are, like ordinary intermittents, more or less distinct, and their intervals more or less complete; so that they may present only the *remittent* form—a form in which an element of continuity seems combined with one of intermittence; or rather there exists here a local affection, of more or less consequence, to which are added, from time to time, periodic phenomena. Such cases are by no means rare. Here, too, quinine is indicated, and will be often found useful. But here, as in the ordinary intermittent, its success will never be so complete as when we have to do with an uncombined intermittent. The condition of the disease will only be simplified, by removing from its permanent form, all that is intermittent or periodical." 569.

### XII. Pulmonary Emphysema considered as a Cause of Death. By Dr. *Prus.*

Notwithstanding the nature of pulmonary emphysema has received much illustration from recent observers, many questions regarding it yet remain in an undecided state. But the author, in the paper before us, confines his attention to the inquiry to what extent may this affection become the immediate *cause of death.* Laennec and Louis do not believe that it tends to shorten life, except through the instrumentality of some other disease; but other celebrated men, as Breschet, Magendie, &c., hold different opinions. Cases shewing that pulmonary emphysema has little effect upon the duration of life are numerous; but these, negative facts as they are, must not be allowed to destroy the value of positive facts proving the gravity and danger of the affection. The author quotes two of these, already published, and adds eight others from his own practice. Facts of this description are of importance, not only for the decision of the true pathology, but also in reference to medical jurisprudence.

Prior to relating his cases, he thus delivers his opinion upon the nature of the disease.

"After an attentive and frequently repeated examination of the subject, it is clear to me that pulmonary emphysema is neither more or less than the infiltration of the air into the intervesicular, interlobular, and subpleural cellular tissue—these three designations characterizing three different stages of the same affection.

"I am ignorant of, and I believe it is difficult to ascertain, what degree of distention the pulmonary vesicles may be subjected to without bursting; and the dilatation of the unbroken vesicles is a thing difficult of proof. M. Bouvier states, that all that has been written upon this subject must be subjected to a renewed examination; and, although I am also of this opinion, I believe I have seen some cases of dilated vesicles, recognizable by the manner in which they were grouped towards the termination of the bronchial ramuscule. I may add my opinion, that a dilatation of the vesicles, which cannot pass such very narrow limits, without inducing a rupture, is a very slight lesion, and is only of importance considered as the commencement of a pulmonary emphysema, *i. e.* the passage of air into the intervesicular tissue. Reasons derived from anatomy, physiology, and pathology, may assist our solution of the question which now occupies us.

"The small size of the vesicles of a healthy lung renders them hardly perceptible, and it is not to be supposed they can reach ten and more times this size without rupture of their fragile walls. Insufflation of a healthy lung performed with moderation, produces a dilatation of its volume which becomes dissipated by the contractility of its own structure. But if the insufflation has been forcible, the dilatation continues permanent. It is then unequal, the air traversing in various directions, and when the insufflation has ceased, only a partial contraction takes place, leaving projections and inequalities on its surface. In the first case the insufflation has distended without rupturing the cells, and in the second, the vesicles have become ruptured, causing an effusion of air into the intervesicular structure, and which, continuing there, has prevented the lung resuming its primitive volume. It is this fact that leads to the practical rule for the performance of insufflation for asphyxia in the gentlest manner. I have seen a case of asphyxia from hanging, in which the vesicles were ruptured and the air infiltrated. M. Devergie, who has filled the office of Inspector of the Morgue for several years, states that, in all the drowned persons brought there, a rupture of the pulmonary vesicles is found to have been caused by the mere forcing back of the air they contain by the water.

"Pathology confirms these views; for, in comparing the emphysematous with the healthy lung, the permanent distention of the one is contrasted with the subsidence of the other under influence of atmospheric pressure. And this arises from the different localities of the air in the two cases. Moreover, the comparative ease with which the air can be pressed from one part to the other proves it is contained in the freely intercommunicating cellular tissue, and not in the air-cells, which communicate only with the bronchial ramifications." 664-6.

Dr. Prus, having detailed the cases and their post-mortem examinations, considers that he may fairly declare death to have been caused by *asphyxia*, occasioned by the pulmonary emphysema, seeing that there was no other means of accounting for its production. The patient, a prey to habitual dyspnœa, which occasionally becomes almost suffocative, and manifesting a greater or less degree of cyanosis, at last dies; and, on examination marked pulmonary emphysema is found, the heart and great vessels are in a normal condition, (except in some instances of slight hypertrophy with dilatation of the right ventricle, which has been proved by Laennec and Louis to be consecutive upon pulmonary emphysema,) no œdema present; the blood in the cavities of the heart black and oily-looking. The vessels of the brain and its membranes gorged with black non-coagulable blood. It is impossible not to seize the connection of the facts of the patient presenting all the signs of defective changes in the blood, and the presence of a disease which in a greater or less degree presented an obstacle to the due hæmatosis. In seven out of the ten cases, the patient was between 56 and 79 years of age, when the cells would have become irregular, dry, and fragile—thus offering every facility for the production of the disease. Frequent opportunities of examining the condition of the parts in broken-winded horses confirm the above conclusions.

Another series of confirmatory observations may be adduced from the fact, that several diseases, which would not otherwise have caused the patient's death, become at once mortal when complicated with this one.

"Thus a Winter does not occur wherein we do not observe in our infirmaries for the aged a great number of patients seized with pneumonia of so very limited an extent, that frequently it only becomes mortal, because a more or less considerable portion of the lung, being affected with emphysema, is unable to effect the requisite changes in the blood. How frequently too have affections of the heart, which could not be said to menace life, except at some distant epoch, become suddenly mortal, owing to this complication." 711.

Upon the connexion of Pulmonary Emphysema with *Asthma,* we have the following observations:—

"To seek, as some do, to explain all the phenomena of asthma by pulmonary emphysema, is to commit a serious error. Those who refuse to be led away by prevailing theories, will recognize in asthmatics three circumstances, which are often united, but may exist separately, viz. the nervous affection, pulmonary emphysema, and bronchitis.

"The neurosis, which is the sole essential condition of asthma, may exist a long time without emphysema or bronchitis. Every physician has seen patients who, after violent paroxysms of asthma, entirely recover perfect freedom of breathing, of motion, and of exercise of all their powers. It is for this neurosis, whether simple or complicated with emphysema or bronchitis, that opium has been prescribed with such constant success by Floyer, Laennec, Louis, and others.

"Pulmonary emphysema, which might be expected to be, and frequently is, the consequence of the deep and violent inspirations of asthmatics, may arise also from other causes, as violent muscular efforts, &c., and need not be accompanied by any of the signs of asthma. M. Louis cites three cases of undoubted pulmonary emphysema, in which the patients suffered from no difficulty of breathing.

"Bronchitis, which, in a great number of cases of asthma, is the sole determining cause of the paroxysm, and in almost all adds singularly to the frequency and intensity of the paroxysm, is not necessarily a companion of either nervous asthma or pulmonary emphysema." 700.

The following are the author's conclusions:—

"1. The seat of pulmonary emphysema is the intervesicular, interlobular, and subpleural cellular tissue. 2. M. Louis is correct in his statement, that when pulmonary emphysema is once produced it always remains. 3. It is generally the case also that the extent and degree of pulmonary emphysema are in direct proportion to its duration. 4. This affection may, by gradually diminishing the hæmatosis, produce a slow death, long foreseen, and constituting the aerian phthisis of Storck. 5. Under other circumstances, it may produce sudden or almost sudden death—these being the cases to which medical legists should especially direct their attention. 6. When, in the absence of any other organic lesion sufficient to account for death, we find, in cases of sudden death, a well developed pulmonary emphysema, a careful examination of the blood should be made. If this is found blackish, fluid, and oily, there is strong reason to believe that death has arisen from asphyxia, produced by the pulmonary emphysema." 721.

### XIII. ON THE POISONOUS PROPERTIES OF SULPHATE OF QUININE. By Dr. *Mélier*.

The timidity with which this medicine was administered at its first introduction, has, according to Dr. Mélier, been succeeded in France by an undue degree of rashness. He especially alludes to the enormous doses (about 100 grains per diem) given by M. Briquet in acute articular rheumatism. Soon after these were recommended, the author and Magendie administered large quantities to several dogs. Most of these animals died, and their lungs were found congested and infiltrated with blood, while their substance in many parts was converted into a spleen-like substance. The blood was in a great measure deprived of its coagulability, the clot softened, diffluent, separating easily from the serum, which remained discolored and thick, holding the colouring matter in solution. It is important, in a therapeutic point of view, to observe that the quinine acted far more energetically on the animals, when given fasting, and when dissolved in sulphuric acid, than when given upon a full stomach, or in its partly soluble condition.

In reference to the effects of too large doses on man, it is to be remarked that many writers had noticed the occasional ill effects of bark, prior to the discovery of quinine, when administered too largely. The author reviews in succession the various cases, giving his authorities, in which quinine has been manifestly hurtful. *Death* is distinctly traced to its use in four cases of articular rheumatism, presenting no especial marks of danger, and occurring within a short period. Delirium and coma, paralysis, epilepsy, pneumonia, hæmaturia, gastralgia and diarrhœa, are among its ill effects. *Deafness*, too, is a pre-eminently common effect. Moderate doses of quinine are indeed sometimes followed by temporary deafness, but after excessive doses it may become incurable.

These accidents are reducible to three categories or degrees. 1. When they affect the nervous system, seeming to be a mere exaggeration of the regular mode of action of the medicine. 2. When the influence is extended to the circulation, whence congestion, pneumonia, hæmaturia, and cerebral mischief. 3. Complete prostration of power, coma, and death.

These observations may throw some light upon the mode of action of the remedy. Formerly, when fears of the ill effects of bark or quinine were expressed, these were directed to the possibility of its producing inflammatory affections of the stomach or intestines; but, although these may be produced they are neither of the same extent or probability as supposed. It is remarkable indeed how large a dose of quinine the stomach will support. It is not therefore the local but the general action of this substance that is to be dreaded. Absorbed into the blood, it changes the constitution of this fluid, depriving it of its coagulability, and acting upon it, when in sufficient dose, in the same manner as many other poisonous agents. Its absorption into the blood and elimination from it, seems to take place very rapidly, as it has been detected in the urine in a very short time;—from which fact the precept may be deduced, not to administer it at too long a period prior to the paroxysm it is directed against. Convinced from the above researches of the danger of these large doses, the author is no less so of their uselessness. Prompt cures have been procured by moderate doses, in the various diseases to which the medicine is applicable, and the having recourse to such extraordinary quantities he attributes to a prevailing rage for excessive doses (perhaps a re-action from former timidity), by which some think everything is to be accomplished.

The author protests against any desire of underrating the utility of quinine, and appeals to a paper of his, which we have already noticed, recommending an extension of its employment to several affections, in which it has not been customary to employ it. He considers it as the most precious of all the agents which have enriched therapeutics. Moreover, in the very affection in question, namely articular rheumatism, he sees reason, from the intermittence which is usually present, to approve of its administration; and adverts to the fact that bark has been recommended by Morton and others for this disease, and the quinine itself employed by others with variable success. He only protests against the scruple and other large doses which tend at once to dishonour the medicine, and to compromise the safety of the patient.

We have left unnoticed the following papers. A Case of Severe Injury to the Brain attended by no serious symptoms, although accompanied by extensive loss of substance. An Essay upon Phthisis in Martinique. A Paper on Hypochondriasis—and one by Dr. Foville upon the Communications between the Cerebrum and Spinal-marrow. The three first present little worthy attention; and the last requires a fuller analysis than our limits now permit. We hope to return to it at a future opportunity.

